# Midwifery Students' Definitions of Normal Labor and Birth: A Study From Five Countries

**DOI:** 10.1111/birt.70056

**Published:** 2026-02-16

**Authors:** International Network Exploring Midwifery Students' Confidence in Physiological Birth, Jalana Lazar, Juliet Wood, Jane Fry, Barbara Baranowska, Urszula Tataj‐Puzyna, Debora Dole, Emma Ritchie, Clare Davison, Cindy L. Farley, Felicity Agwu Kalu, Maria Healy, Maria Węgrzynowska

**Affiliations:** ^1^ Frontier Nursing University Versailles Kentucky USA; ^2^ Department of Midwifery and Health Sciences Bournemouth University Bournemouth UK; ^3^ Department of Midwifery Centre of Postgraduate Medical Education Warsaw Poland; ^4^ School of Nursing Georgetown University Washington DC USA; ^5^ School of Nursing and Midwifery Edith Cowan University Joondalup Western Australia Australia; ^6^ Edith Cowan University Joondalup Western Australia Australia; ^7^ Charles Darwin University Darwin Northern Territory Australia; ^8^ Nurse‐Midwifery/WHNP & WHNP Programs, School of Nursing Georgetown University Washington DC USA; ^9^ School of Nursing and Midwifery Queen's University Belfast, MBC Belfast UK

**Keywords:** maternal health, midwifery education, midwifery students, neonatal health, normal labor and birth, physiological labor and birth

## Abstract

**Introduction:**

Internationally, many women and birthing people are receiving maternity care interventions as a routine with no medical indication for their use. This medicalized environment influences midwives' practice and, by extension, midwifery students' clinical learning experiences, gaining knowledge and confidence in facilitating normal labor and birth. To educate midwifery students in the provision of high‐quality maternal and newborn care, it is important to explore their understanding of normal labor and birth, as this underpins their philosophy of care and will inform their future practice.

**Methods:**

An online survey was developed from a review of current evidence. Following ethical approval, the survey was disseminated at midwifery education programs across five countries—Australia, England, Northern Ireland, Poland, and the United States—between February 2019 and December 2023.

**Results:**

In total, 664 midwifery students responded to the open‐ended question: “What do you consider the phrase ‘normal labour and birth’ to mean?” Reflexive thematic analyses resulted in seven themes, representing the key components of the midwifery students' definitions of normal labor and birth. These included: “Vaginal birth with no or minimal intervention in labour; Spontaneous onset of labour; Respectful women‐centered care; The concept of normal is disputed; The midwife's role; Birth outcomes define normality; and Environment and freedom to move.”

**Conclusions:**

Despite some differences in maternity care and midwifery education across the five countries, midwifery students develop a comparable understanding of normal labor and birth gained through their education and clinical experiences, which has implications for the future of midwifery practice and identity.

## Graphical abstract

The image brings together the seven aspects considered most important in defining ‘normal/physiological birth’ according to research carried out among 664 student midwives across five countries.

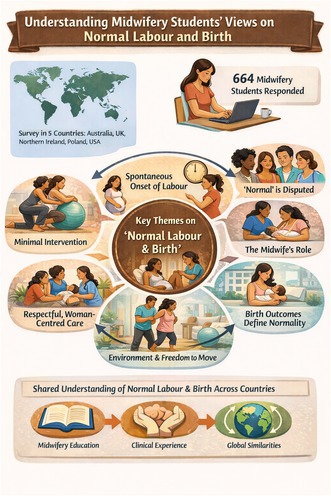

**Abbreviations**
AU: Australia
EN: England
ICM: International Confederation of Midwives
NI: Northern Ireland
PO: Poland
UK: United Kingdom
US: United States of America
WHO: World Health Organization

## 1 Introduction

Midwife faculty from Australia (AU), England (EN), Northern Ireland (NI), Poland (PO), and the United States of America (US) expressing an interest in midwifery students' confidence supporting normal, physiological birth connected at an international research conference in England in June 2019. They shared concerns that the medicalization of birth may lead to devaluation and deskilling of essential midwifery knowledge and practices. Global birth trends showed declining rates of spontaneous labor and vaginal birth and rising rates of medical interventions such as induction of labor, epidural analgesia, and episiotomy [1, 2, 3, 4, 5, 6, 7, 8]. In each of our five countries, the cesarean birth rate hovers at or above a third of all births, and the rate of spontaneous vaginal birth reflects a decline, with only half of births recorded as physiological vaginal births in Australia, England, Northern Ireland, and the United States and even fewer in Poland [1, 2, 8, 9, 10].

Where does this leave the midwife, whose professional role is often defined in relation to supporting normal physiological childbirth, and the student learning these skills? The International Confederation of Midwifery (ICM) position paper on Keeping Birth Normal states:

ICM's midwifery philosophy emphasises pregnancy, birth and postpartum as normal and profound life experiences and stresses the role of the midwife in keeping them normal. Midwives optimise normal physiological processes and support safe physical, psychological, social, cultural and spiritual situations, working to promote positive outcomes and to anticipate and prevent complications. [11]

Midwifery care has been globally endorsed as an essential element of quality maternal and newborn care; it is associated with improved outcomes and reduced disparities in care [12, 13, 14, 15, 16]. Midwives in each of our five countries historically and currently support physiological labor and birth, defined by ICM as a “process whereby the woman or gender diverse person commences, continues, and completes labour with the infant being born spontaneously at term, in the vertex position, and without surgical, medical, or pharmaceutical intervention” [11]. The WHO position on what constitutes a “normal labour and birth” reflects the ICM's definition but additionally states, “after birth the mother and baby are in good condition” [17].

Midwifery education varies within and between each of the countries in our study, but all registered midwives meet the ICM criteria for educational preparation and core competencies of a midwife. In England, Northern Ireland, and Australia, midwives are among the lead maternity professionals for healthy childbearing people with straightforward pregnancies; in the United States and Poland, midwifery is often subordinate to obstetrics. US midwives attended only 12% of births whilst midwives in Poland are being largely excluded from providing antenatal care [18, 19]. Yet in each country, national standards of midwifery competence and care reflect the ICM in their professional association documents [20, 21, 22]. All the researchers in this study are midwifery educators, teaching curricula designed to prepare midwives to meet standards of care that endorse physiological labor and birth and align with the ICM and WHO definitions.

How midwives define the core responsibility of their profession—the safe conduct of labor and birth—influences their approach to care. Adopting a definition of birth as a normal, physiologic process leads to support for the spontaneous work of the body and to work in partnership with the parturient's preferences, hopes, and fears. Alternately, defining birth as a potential pathology justifies pre‐emptive procedures designed to control labor and birth processes. As such procedures have become normalized in today's highly interventive birth environments, what definition of birth is the next generation of midwives developing? Social learning theory suggests that role models and authentic experiences are powerful influences on learners' knowledge and confidence and can mediate behavior [23]. Cognitive processing and the reciprocal relationship between environment and personal factors also affect the influence of social learning on performance.

Given that midwifery students are being educated in birth environments that reflect declines in physiological labor and birth, effectively enabling students to develop skills and knowledge to support and care for women and gender diverse people during normal labor and birth is challenging. Some student midwives complete their clinical education without witnessing or caring for a person experiencing spontaneous labor and birth [24]. It is important to understand what the terms normal labor mean to students, given the shifts in birth trends and the implications for midwifery students' learning and confidence around physiological birth. This study explored student midwives' definitions of normal labor and birth in five countries. The authors recognize that not all pregnant people identify as women, and have used inclusive terms where appropriate; however, in keeping with good research practice, we reflected the language used by the study participants, who primarily used gendered terminology.

## 2 Materials and Methods

### 2.1 Design and Survey

Thematic content analysis was applied to the qualitative data obtained from a survey of midwifery students exploring how they define normal labor and birth. This data was in response to 1 open‐ended question of a 14 question survey exploring student midwife confidence to support normal labor and birth [25]. The survey was developed following a review of current evidence. Face and content validity was ascertained in each country by midwifery educators and students. The survey was pilot tested and minor edits were undertaken. Some word choices were adapted to fit local language and maternity service provision in participating countries. The Polish questionnaire was translated bidirectionally to assure clarity and comparability of the survey across the English‐speaking countries. Data collection was staggered over several years to accommodate international researchers and midwifery program participation and varying international educational timetables. This paper presents data relating to the free text open‐ended question from the survey: “What do you consider the phrase ‘normal labour and birth’ to mean?”

### 2.2 Participants

A convenience sample strategy was used. Students eligible to participate were enrolled in a midwifery education program at a single university in England, Northern Ireland, and the US, two universities in Australia, and at three in Poland during the timeframe of the study (see Table S1).

### 2.3 Data Collection

The data were collected within each country at different time points between 2019 and 2023, via an online anonymous survey link emailed to students in the participating cohorts.

### 2.4 Ethics

Ethical approval was obtained by all participating universities following national and university policies. Participation in the study was voluntary. Criteria for the study were explained and completion of the survey indicated consent. Student responses were anonymized, thus potential risks of student vulnerability were mitigated.

### 2.5 Research Question and Analysis

The question “What do you consider the phrase ‘normal labour and birth’ to mean?” invited a free‐text response. A process of reflexive thematic analysis was undertaken by each country team using a realist semantic approach to capture the essence of the responses without imposing a particular conceptual framework or researchers' philosophical assumptions on the text [26]. Initially, the researchers read through the data several times to familiarize themselves with the content. Small sections of text were then coded, and these were then grouped as themes were generated. This was an iterative process by each researcher and between researchers, first at country level and then between countries. The themes identified the key components which defined “normal labour and birth” to the respondent. Some themes were common to all five countries, whilst others were only identified and described in several countries. The positionality of the research team is that supporting physiologic labor and birth is an essential skill for students to learn and is constrained in today's over‐medicalized birthing environments.

## 3 Results

A total of 664 midwifery students completed the survey. Participation rates by country were as follows: Poland 219 (33%), England 181 (27%), Northern Ireland 106 (16%), United States 85 (13%), and Australia 73 (11%). Several themes were found to be universal within the students' definitions in all five participating countries, whereas other themes appeared in most but not all countries. The data centered their definition of “normal labour and birth” as follows:

- “Vaginal birth with no or minimal interventions in labour” (*all*);
- “Spontaneous onset of labour” (*all*);
- “Respectful women centred care” (*all*);
- “The concept of normal is disputed” (*all*);
- “The midwife's role” (*AU, EN, US*);
- “Birth outcomes define normality” (*AU, PO, US*);
- “Environment and freedom to move” (*AU, EN, NI*).

### 3.1 Vaginal Birth With No or Minimal Interventions in Labor

Most students understood normal birth to be physiological vaginal birth with “no or minimal” interventions. Students indicated that physiological vaginal birth was normal. They repeatedly used the words, “no intervention” or “minimal intervention” as a qualifier to their statements on vaginal or physiological birth. It is not clear from the student data what constitutes a “minimal” intervention. One student stated a minimal intervention “allow[ed] the natural process of labour and birth to happen (NI)*”*, another qualified minimal intervention as “IV fluids and AROM (US)”, whereas others referred to “little intervention other than some pain relief (EN)”, “vaginal birth without artificial hormones (EN),” and “no augmentation of labour (US).” Students based in Australia and England mentioned the third stage of labor as another area where intervention should be kept to a minimum. Conversely, one student stated, “Normal labour and birth might include CTG monitoring, epidural use, syntocin augmentation and active third stage management (AU).”

### 3.2 Spontaneous Onset of Labor

A key component of the students' definitions of normal labor and birth referred to how labor was initiated. Midwifery students demonstrated an appreciation of the benefits of birth beginning physiologically in their definitions.

- “Spontaneous labour with the undisturbed arrival of the newborn” (*PO*);
- “Spontaneous onset of labour, minimal interference, no augmentation of labour, normal vaginal delivery” (*US*);
- “Normal birth is respecting physiology and autonomy, its spontaneous and undisturbed” (*AU*);
- “I understand the process, the hormones involved, what helps increase oxytocin, I think by definition this means no induction/augmentation of labour” (*NI*).

### 3.3 Respectful, Woman‐Centered Care

Many definitions emphasized respectful and empathetic care as what makes birth normal. These definitions included terms referring to respect, accepting the woman's decisions, and considering her needs and desires. Some of these definitions also acknowledged minimizing complications or interventions as part of what constituted normal birth. For example, “normal birth” was “a birth where the woman gets what she wanted and needed out of it with little interference (EN)” and that “normal labour and birth is the normal physiological process, it should be woman focused with a holistic approach to wellbeing (AU).” Others stated “One where a woman is empowered to have her baby without any unnecessary interventions (NI)” and “A woman is free to labour and birth in her own time, on her own terms (AU).”

Some of these definitions even stressed that irrespective of the mode of labor or the types of interventions, what makes birth normal is respectful, empowering care and honoring women's needs. For example, normal birth was defined as

- “A birth that the patient is comfortable with, understands, and feels empowered in” (*US*);
- “Supporting a labour based on the woman's wishes whether that is an active labour or a labour with an epidural” (*EN*);
- “Normal birth means that there is a nice atmosphere, the staff shows support, help and advice, and the woman in labour knows what she wants and cooperates willingly with the staff. Normal birth does not have to end ‘naturally’, without anaesthesia or an episiotomy. For me, a normal birth means love for the baby and trust in the staff” (*PO*).

### 3.4 The Concept of Normal Is Disputed

One of the themes that emerged was the disputed concept of the term “normal.” Some of the students questioned the validity and appropriateness of the term “normal.” They disputed the word itself and disliked the way in which it detracted from the individuality of each birth and person and stigmatized certain events or outcomes.

- “Normal is not a word I ever like to use, as what is normal?” (*EN*);
- “Normal is a phrase that can be misconstrued into many different meanings” (*AU*);
- “It is a tautology. All labour and birth is normal” (*US*);
- “Normal birth is subjective and unique to each woman” (*AU*);
- “I avoid using this term with patients as I believe ‘normal’ is a relative term and that birth support should be individualized” (*US*);
- “I think it is hard to define as to women it means different things that it does to us as midwives” (*EN*);
- “In my opinion, this phrase should not be used because it is stigmatising. Mums after a natural childbirth as well as after a <caesarean birth> are just as valuable” (*PO*).

Confusion around defining normality birth was recognized in the student data. Normal birth is “one that progresses naturally, without intervention and at its own pace. Having said that I have seen lovely normal births, even if the labour has featured an intervention such as induction or continuous CTG (EN).”

### 3.5 The Midwife's Role

Students in three countries (AU, EN, US) identified the midwives' role to primarily align to the individual's preferences and condition. If the woman or birthing person has unique and individualized ideas on what their normal birth is, then it is the midwife's role to support that, even if some ideas are atypical. “Having faith in the physiology of birth (US)” and “trust in the natural process of birth and belief that birth is a normal part of the human experience, not a medical condition (US)” indicated that the faith or trust that the midwife has in the process is a determinant in the normality of birth. This theme was reinforced. “A midwife's role is to enhance normality during the labour process (AU)” and to “believe in the importance of physiological birth (AU).” The attending midwife's confidence and advocacy in supporting and facilitating normal birth were key components of their role in maternity care. The midwife's role in normal birth was “giving encouragement and support to the woman and believing in her ability and ensuring she believes in herself and her body (EN)” and “listening and watching the woman, letting her listen to her body and midwives respecting that (EN)”. Similarly highlighted was how the midwife can “support a woman during labour (NI),” “optimise service user experience…and understand and build a relationship with women and their families (NI).”

### 3.6 Birth Outcomes Defines Normality

In three countries (AU, PO, US), students' definitions of normal birth included the birth outcome, and often the absence of birth complications. Outcomes included healthy baby and healthy mother, term births, vaginal births, absence of labor interventions, or included a focus on the safety of the birth as part of the definition. Normal birth is:

- “Least amount of medical or invasive interventions as possible to have a good/healthy outcome for both mom and baby” (*US*);
- “Physiological progression through the stages of labour without complications such as shoulder dystocia, postpartum haemorrhage” (*US*);
- “Minimal trauma to mother or baby” (*AU*);
- “Birth at term, (37–42 weeks) with no complications, mother and the baby after the birth feel fine and the baby is healthy” (*PO*).

This indicates that students are reluctant to assign normality to the process of birth without considering the outcome. Normal is then a retrospective description applied to labor and birth.

In the Polish dataset, a minority of students' responses to the definition of “normal birth” focused only on the route of childbirth, excluding references to the medicalization, birth environment, outcomes, or the role of the midwife or any other healthcare professional. The frequency of these answers was considerably higher for the Polish data than for any other country participating in the study. This included responses such as “[normal birth is] vaginal birth (PO)” and “[normal birth is] birth by natural means and forces (PO).” (The expression “by natural means and forces” is a direct translation from of the Polish phrase “poród siłami i drogami natury.” This is a popularly and officially used expression for all vaginal births [excluding forceps and vacuum]).

### 3.7 Environment and Freedom to Move

Students in three countries (AU, EN, NI) put emphasis on the environment and the opportunity to move.

- “The ability to move around freely without being restricted to the bed and birth in whatever position the mother wants” (*EN*);
- “Using movement and following the instinct of the woman, having her birth on her timeline, not the hospital's” (*AU*);
- “The woman is able to move freely and listen to her body” (*AU*);
- “Labour in the vertical position, taking into account the patient's opinion and needs in terms of choice of position, conditions of birth and her expectations” (*PO*).

Although some Polish midwifery students referred to the freedom of movement in their definitions of normal birth, very few mentioned the birth environment. This may be linked to the limited alternatives to hospital birth the Polish maternity services currently offer. As of July 2024, there were only two alongside hospital midwife‐led birth centres, both located in the capital city of Warsaw. There are no freestanding midwife‐led birth centres and home birth is not publicly funded [27].

## 4 Discussion

Normality is a disputed concept among midwifery students. The concept of “normal” is a social construct—it can mean “expected,” “usual,” “natural,” or “average.” The circumspection around the concept of normality reflects realities of midwifery practice in the clinical setting. The reasons for this are multifactorial and interconnected, depending on whether there is intervention in the physiological process, to what degree, and for what indication; the structure and environment of care; the philosophy and power dynamic of the woman and family, the practitioner or the healthcare system; and whether one is making the definition prospectively or retrospectively. The theme “The concept of normal is disputed” indicates the need for a better descriptor. Are all births “normal” as noted by one respondent or are no births “normal”? A critique of midwifery care is the over‐normalization of birth, leading to delay in enacting needed interventions or consultations. However, whilst this may occur in isolated cases, it is not supported by midwifery care process and outcomes data [13]. There is consensus among the students in all five countries that normal birth should include “no or minimal interventions,” yet there is no clear agreement in the data as to what constitutes minimal intervention. Interventions in labor and birth are commonly described as “caesarean section, operative vaginal delivery, episiotomy, epidural anaesthesia and oxytocin augmentation” [21, 28]. Other interventions include artificial rupture of membranes (AROM), IV fluids, continuous fetal monitoring, scalp electrodes, intrauterine pressure catheters. The steady rise in frequency of one particular intervention, elective induction of labor, has not escaped notice in students' definitions. “Spontaneous onset of labour” as a recurrent theme among their definitions of normal childbirth reflects an understanding of labor induction as a disruption of the biologically complex process of childbirth [29].

Furthermore, *respectful maternity care*, a tenet of midwifery, emerged as a theme in midwifery students' definitions of normal birth. Midwifery students may perceive describing low intervention vaginal births as “normal” could construe other birth as “abnormal” which is not a desirable label and may feel disrespectful to women and thus in opposition to supporting respectful maternity care. The World Health Organization (WHO) advocates implementing respectful maternity care that values women's individual, cultural, personal, and medical needs as an essential strategy for universal access to high‐quality care [17]. The right to respect, equity, and autonomy in decision‐making are central to women's health [30, 31, 32]. Respectful maternity care (RMC) is considered a practical manifestation of work within the broader philosophy of midwifery, which assumes being “with the woman” [33]. Supporting normal birth simultaneously supports respectful maternity care, which is reflected in student responses that highlight RMC, but also indicates students may need support implementing both normal birth and RMC.

Further influencing student definitions of normal birth is the reality that obstetric intervention is common [34]. If midwives, who are recognized experts in normal birth, exclude themselves from births that include obstetric interventions, then they are limiting their ability to support a great number of women and birthing people, which runs counter to midwifery philosophy of advocating for a “midwife for every woman” [35, 36]. As rates of straightforward physiological birth decline globally, how do midwives, as the professional guardians of physiological birth, maintain a role or relevance if not by expanding the definition of what physiological birth entails, taking into account changes in social conventions and sensitivities? In acknowledging the maternal desires and collaboration in the definition of physiological birth, students reflect exposure to the vital role of the midwife as advocate and also the challenge of reconciling maternal desires and institutional constraints [37]. If midwives exist as advocates for women and women are influenced in an increasingly risk‐based society to view “normality” in birth as outcome‐based exclusively, midwives may find their definitions of normality in childbirth a barrier in providing care to the women for whom they are advocating. Students coming to terms with the descriptor of “normal” as applied to birth may signify some of the changing views of new generation midwives and society in general, which may in future lead to a reevaluation of the term and the way in which it is used throughout midwifery textbooks and curricula globally [38]. Student definitions that focused on birth outcomes also hazard diminishing the value of the physiological process of birth, which can also call into question the value of the midwifery role in supporting physiology. This is important given our increasing understanding of the underappreciated benefits of vaginal birth and the overlooked risks associated with repeat cesarean birth [39, 40, 41].

An element unique to Poland was found in the high rate of responses focusing on the route of birth. This may indicate a dichotomy in the way childbirth is perceived in Poland, both in clinical settings as well as in popular culture. Births are reported either as “vaginal” or “caesarean sections” indicating “vaginal” as “natural” and “caesarean section” as “medicalized.” There are no official data on the use of oxytocin, epidural, place of birth or episiotomy within Polish maternity care services. The only officially reported data on the medicalization of birth in Poland is the number of cesarean sections. In Polish popular discourse, the word “natural” is often used as a synonym of “vaginal” birth.

The findings of this study are cautiously optimistic for the profession of midwifery to maintain its claim as guardians of physiologic birth. The themes of “Vaginal birth with no or minimal intervention in labour; Spontaneous onset of labour; Respectful women‐centered care; The midwife's role; and Environment and freedom to move” are supportive of such a professional role identity. However, the theme “Birth outcomes define normality” is often used to rationalize intervention before an indication for use develops. Social learning theory suggests that confidence can be a mediating variable in performance when facing disconfirming circumstances, such as midwives face daily in over‐medicalized birth environments, and is important to develop in students [23, 25]. Global outcome data continually validate the midwifery philosophy and model of care as safe and satisfying [13, 14, 16].

### 4.1 Strengths and Limitations

Despite varying contexts, aspects of students' definitions of normal birth were universally expressed. Some themes, however, only arose in certain countries. This may reflect cultural differences in midwifery practice and education between countries. Of note, there is particular sensitivity around the word “normal birth” in the UK context [42]. Two countries of our study are part of the UK, potentially over‐representing this view in our data. The possibility of multiple interpretations of the Polish words for “physiological birth” could affect student responses. For example, the word “natural” may be more commonly used than the word “normal” in relation to labor and birth in Poland.

While normal and physiological are descriptors often used interchangeably, they are not the same. Normal birth generally refers to a vaginal birth without major medical interventions, while physiological birth specifically emphasizes the natural process where the body is supported to follow its own rhythm, minimizing interference in both the labor and birth stages. Our study asked about normal birth as more likely to be sensitive to social influences.

Data were collected during the height of the COVID‐19 pandemic and the immediate postpandemic timeframe when many supportive practices in birth care were suspended, thus potentially altering perceptions of normal birth, particularly by students entering the field of midwifery at this time.

### 4.2 Recommendations

Settling on a student understanding of normal childbirth is in itself not straightforward. Notably, a recent systematic review [43] demonstrated that practicing midwives reflect similar conflicts and concerns around definitions of normal birth as those expressed by the students in this study, suggesting an existential and not merely experiential element to the interplay of midwifery and defining normal birth.

Didactic teaching including extensive exposure to evidence on the process and public health benefits of physiological birth plays a role in supporting student confidence that physiological birth is both desirable and possible. The clinical setting in the current context may support or countermand that teaching. Future research exploring how student definitions of physiological birth affect practice and how views of birth evolve over time as students graduate and begin their careers would contribute to our knowledge. Furthermore, professional power differentials for midwives might negatively impact student commitment to a physiological approach to care. The importance of midwifery students being exposed to physiological birth during their training through observation and participation in community midwifery settings, where the rates of physiological birth are high and the physical environment is supporting physiological processes, is strongly supported in our data as well as in social learning theory. Midwifery students should attend undisturbed physiological births during their training and observe examples of physiological birth in simulation training with concomitant reflection and discussion.

## 5 Conclusion

This study of students' definitions of normal birth in five countries highlighted the challenges around defining normal birth in the current climate of common routine interventions. Our data suggest that students are evolving definitions of birth influenced by the increased medicalization of childbirth. However, students are at a transitional phase in their professional identity formation. Acknowledging and addressing challenges to supporting physiological labor and birth at the curricular and clinical level with midwifery students is essential. Providing ample opportunities for students to practice respectful maternity care and support physiological birth are necessary to continue to promote and protect the art and science of midwifery education and practice.

## Funding

The authors have nothing to report.

## Ethics Statement

Ethical approval was initially obtained from the Research Ethics Panel of Bournemouth University (REC reference: 24299). Each additional participating country received ethical approval as per their national standards.

## Conflicts of Interest

The authors declare no conflicts of interest.

## Supporting information


**Table S1:** Participating midwifery education program characteristics.

## Data Availability

The data that support the findings of this study are available from the corresponding author upon reasonable request.
