# Supplementary material for: Midwifery Students' Definitions of Normal Labor and Birth: A Study From Five Countries
Source: Birth. 2026 Feb 16;53(3):596–603. doi: 10.1111/birt.70056 (PMC13471783; doi:10.1111/birt.70056)
Supplement: Supplementary file 1 [file BIRT-53-596-s001.docx]

**Supplementary Table 1. Participating midwifery education program characteristics**

| **Country** | **Participating**  **universities** | **Degrees offered & length of program** | **Entry qualification** | **International students & countries of origin** | **Age range of students** |
| --- | --- | --- | --- | --- | --- |
| EN | AA University | BSc (Hons) Midwifery (3 years) | Post-secondary with proper academic qualifications | Several (ex. Poland, Spain, France, USA, South Africa, Ireland, Nigeria) | 18-54 |
| NI | BB University | BSc (Hons) Midwifery (3 years)  MSc Midwifery (2 years) | Post-secondary with proper academic qualifications | Kingdom of Saudi Arabia, USA | 18 - 50 |
| PO | CC University | BSc (Hons) Midwifery (3 years)  MSc Midwifery (2 years) | Post-secondary with proper academic qualifications | None | 18-50 |
|  | DD University |  |  |  |  |
|  | EE University |  |  |  |  |
| AU | FF University | Bachelor of Nursing and Midwifery, 4 years full time  MSc of Midwifery, 1.5 years full time | Post-secondary with proper academic qualifications | None | 18-50 |
|  | GG University | Bachelor of Midwifery, 3 years full time |  |  |  |
| US | HH University | MSc  (2 years, 4 months)  Doctor of Nursing Practice (2 years, 8 months) | Registered Nurse with a Bachelor’s degree | None | 25-40 |

BSc (Hon) – Bachelor of Science with Honours; MSc – Master of Science
